# Residual based anomaly detection framework for variant caller dispatch in DNA sequencing data

**DOI:** 10.3389/fmicb.2026.1877028

**Published:** 2026-09-08

**Authors:** Shenjie Wang, Yuhang Li, Kai Quan, Jiayin Wang

**Affiliations:** 1 Department of Respiratory Medicine, The Second Affiliated Hospital of Xi’an Jiaotong University, Xi’an, China; 2 School of Computer Science and Technology, Xi’an Jiaotong University, Xi’an, China; 3 Shaanxi Engineering Research Center of Medical and Health Big Data, Xi’an Jiaotong University, Xi’an, China

**Keywords:** anomaly screening, DNA, genomic windows, sequencing data, variant-caller dispatch

## Abstract

Reliable and scalable variant analysis is an enabling component of genomic studies involving microbial communities, host-associated microorganisms, and their hosts, and may support future investigations of genetic heterogeneity within symbiotic systems. Widely used workflows incorporating BWA and GATK provide standardized default processing routes, but their performance may vary across genomic regions containing repetitive sequences, complex structures, or atypical local sequence characteristics. Applying a context-aware software-recommendation procedure to every genomic region, however, can substantially increase computational demand. Here, we present LSTM-EWMA, a screening-and-dispatch framework designed to identify genomic regions that should be considered for specialized downstream evaluation. The framework represents ordered genomic regions as a sequence of feature vectors, uses a Long Short-Term Memory (LSTM) network trained exclusively on predefined in-control (IC) regions to model baseline patterns, and applies an Exponentially Weighted Moving Average (EWMA) control chart to standardized prediction residuals. Regions exceeding prespecified control limits are operationally labeled as out-of-control (OC) and designated as candidates for downstream software recommendation, whereas unflagged regions remain on the default processing path. These labels describe computational workflow states and do not independently confirm genomic variants or biological abnormalities. Using simulated sequencing data derived from the human reference genome as an initial methodological benchmark, LSTM-EWMA distinguished predefined OC regions from IC regions while maintaining a low observed false-alarm rate under the evaluated settings. These findings support the feasibility of the dispatch strategy within the current simulation design and provide a defined basis for subsequent evaluation in microbial, metagenomic, and host-associated sequencing contexts. With further validation across taxonomically diverse and biologically characterized datasets, LSTM-EWMA could support scalable variant-analysis workflows for microbial community and symbiosis research. The source code is publicly available at https://github.com/Icarus200110/Lstm-EWMA.

## 1 Introduction

Reliable identification of genomic variants provides one technical foundation for characterizing biological heterogeneity across human genomes, microbial genomes, and host-associated microbial systems (Partida-Martinez et al., 2007; The Human Microbiome Project Consortium, 2012). In microbial symbiosis research, genomic variation can complement taxonomic, functional, and metabolic measurements, and integrating these evidence layers can support more comprehensive investigations of interspecies interactions, host responses, and ecosystem resilience. Host-associated systems are shaped by interacting nutritional, microbial, immune, and epithelial processes. For example, the interplay among nutrition, gut microbiota, and host immunity can influence responses to enteric infection (Huo et al., 2025); dietary galacto-oligosaccharide supplementation has been reported to alter gut microbial composition alongside intestinal and growth-related outcomes in weaned piglets (Wang et al., 2025); and cellular experiments have shown that the food-associated mycotoxin deoxynivalenol can disrupt intestinal tight-junction proteins through specific endocytic and signaling pathways (Li et al., 2021). Mathematical and artificial intelligence-based models are also increasingly being investigated for data integration and prediction in precision animal nutrition (Hu et al., 2025). Although these studies address different biological questions, they collectively illustrate the heterogeneity of host-associated systems and the growing demand for scalable analytical methods.

Next-generation sequencing has expanded the genomic investigation of vertebrate-associated microbial systems (Ley et al., 2008) and supported the widespread adoption of standardized genome-analysis workflows, including BWA for sequence alignment (Li and Durbin, 2009) and GATK for downstream variant processing (McKenna et al., 2010). However, the performance of a single default workflow may vary across genomic regions with different GC content, repetitive sequences, local structural complexity, or other atypical sequence characteristics (Treangen and Salzberg, 2011). These region-dependent considerations are especially relevant to microbial and metagenomic sequencing, which involves extensive sequence diversity, mixtures of closely related organisms, and uneven organismal abundance that can complicate alignment and downstream variant interpretation (Andreu-Sánchez et al., 2021). Comparative pipeline studies and integrated benchmark call sets further underscore the importance of carefully defined evaluation data and reference variants for reliable assessment of variant-analysis performance (Hwang et al., 2015; Zook et al., 2014). Microbial communities can exhibit substantial taxonomic variability and functional redundancy, while certain keystone taxa may exert disproportionately large effects on microbiome structure and functioning (Banerjee et al., 2018; Louca et al., 2018). These ecological characteristics illustrate the biological heterogeneity of microbial systems and motivate the development of analytical methods capable of resolving variation across community members and genomic contexts. Reliable and computationally efficient processing of heterogeneous genomic regions is therefore relevant to a broad range of sequencing studies, including prospective applications in microbial and host-associated systems.

Deep learning has been applied to variant calling and a broader range of context-aware genomic prediction tasks (Eraslan et al., 2019; Poplin et al., 2018). Such methods generally use regional genomic features to inform computational inference (Zou et al., 2019), providing a methodological foundation for region-specific software recommendation according to localized genomic characteristics. In this setting, regional features can be extracted and used to identify a potentially suitable variant-calling strategy. However, calculating multidimensional features and evaluating software suitability across all candidate regions of a large sequencing dataset can substantially increase computational demand. Accordingly, this study focuses on an upstream resource-allocation question that complements variant calling itself: whether genomic regions can first be screened so that a resource-intensive recommendation procedure is invoked only for regions meeting predefined dispatch criteria.

To formulate this screening problem, we represent consecutive genomic regions as an ordered sequence of feature vectors. Genomic order is used here as a computational sequence without implying biological time, thereby permitting the adaptation of sequential monitoring methods to region-level genomic data. In statistical process control, monitoring procedures are used to distinguish observations that remain consistent with a reference process from those that exhibit a detectable shift (Freeman et al., 2021). Long Short-Term Memory (LSTM) networks can model nonlinear dependencies within ordered data (Van Houdt et al., 2020), whereas Exponentially Weighted Moving Average (EWMA) control charts accumulate information across successive observations and can be used to detect persistent deviations from a reference state (Lucas and Saccucci, 1990). Hybrid approaches combining learned predictors with statistical monitoring procedures have consequently been explored in several sequential anomaly-detection settings (Hundman et al., 2018; Malhotra et al., 2015; Niu et al., 2020). This complementarity motivates the present framework and provides a methodological basis for evaluating sequential residual monitoring on region-level genomic data.

Inspired by this rationale, we propose LSTM–EWMA, a two-tier dispatch framework that combines LSTM-based prediction with EWMA monitoring of prediction residuals. Within the proposed workflow, regions assigned to the baseline processing path are operationally designated as “in-control” (IC), whereas regions identified for specialized software recommendation are designated as “out-of-control” (OC). These terms provide clearly defined computational labels for distinguishing the two processing paths and enable the dispatch decision to be evaluated independently of subsequent biological interpretation. An LSTM model is trained using IC-region data to learn the sequential patterns represented in the baseline feature set. During region-by-region evaluation, the model generates prediction residuals, which are standardized and monitored using an EWMA statistic. An EWMA alarm therefore functions as an operational signal for activating the downstream recommendation procedure, establishing a transparent connection between residual monitoring and computational resource allocation.

The intended workflow retains the default variant-calling strategy for regions without an OC alarm and activates the more computationally demanding recommendation module for alarmed regions. In this way, the framework aims to reduce the number of regions requiring specialized evaluation while retaining the ability to identify departures from the learned baseline under the assessed conditions. The present study evaluates this screening strategy using simulated sequencing data derived from the human reference genome, providing a controlled methodological benchmark for examining the feasibility, operating characteristics, and component contributions of the proposed framework. This initial evaluation establishes a reproducible foundation for subsequent transfer to microbial, metagenomic, and host-associated sequencing contexts. It also defines a clear pathway for extending the framework through validation across diverse microbial species, complex community compositions, multiple sequencing platforms, and experimentally characterized symbiotic systems. With such continued development, LSTM–EWMA could serve as a scalable computational component for allocating variant-analysis resources in studies of microbial communities and host-associated systems, thereby supporting future investigations of genomic heterogeneity in microbial symbiosis.

## 2 Materials and methods

### 2.1 Overall framework

**FIGURE 1:**
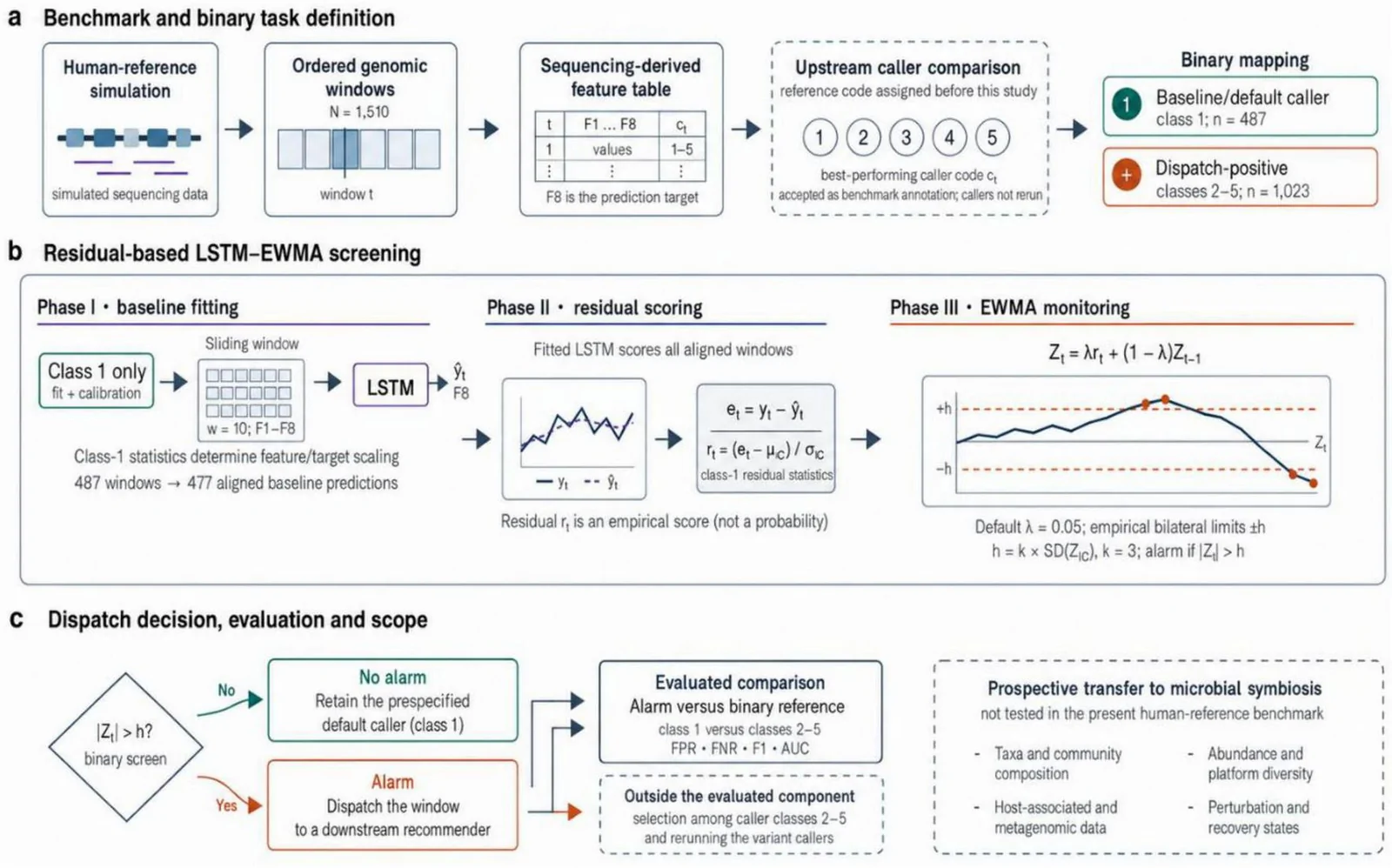
Scope of the Long Short-Term Memory (LSTM)–Exponentially Weighted Moving Average (EWMA) screening component. **(a)** Benchmark and binary task definition. **(b)** Residual-based LSTM–EWMA screening. **(c)** Dispatch decision, evaluation, and scope. The component evaluated in this study distinguishes windows assigned to the baseline caller (code 1) from those routed to a downstream caller-recommendation procedure (codes 2–5). Selection among the callers represented by codes 2–5 occurs at the downstream stage of the proposed workflow and is outside the scope of the present evaluation.

Figure 1 defines the scope of the evaluated component. First, an LSTM is fitted to windows assigned to the baseline/default-caller class. Second, predictions are produced for the ordered feature table and residuals are standardized with class-1 statistics. Third, an EWMA statistic is compared with empirical bilateral limits. A signal serves as an operational request to invoke a downstream recommendation module. The experiments assess agreement with the binary reference label (class 1 versus classes 2–5), providing a controlled evaluation of screening before caller selection and variant-caller rerunning in the downstream stage.

### 2.2 Benchmark and problem formulation

The internally generated benchmark was derived from simulated sequencing data based on a human reference genome and contains *N* = 1,510 ordered genomic-window samples. The working table has nine columns: eight numerical features extracted from the sequencing data (F1–F8) and one reference optimal-caller code. Because the retained analysis code identifies the features by column position rather than by semantic names, we retain F1–F8 to preserve direct correspondence with the implemented analysis. F8 is the regression target; feature windows containing F1–F8 from preceding samples are the LSTM inputs. The ninth column contains l_t_ ∈ {1, 2, 3, 4, 5}. Class 1 denotes the baseline/default caller, and classes 2–5 denote samples for which an alternative candidate caller was labeled optimal after all candidates were run and compared upstream.


$${\text{x}}_{\text{t}}=\left[{\text{x}}_{t,1},\ldots ,{\text{x}}_{t,8}\right],{\text{y}}_{\text{t}}={\text{x}}_{\text{t},8},{\text{l}}_{\text{t}}\in \left\{1,2,3,4,5\right\}$$

There are 487 class-1 samples and 1,023 class-2-to-5 samples. All 487 class-1 samples were used to fit the feature and target StandardScaler objects, construct LSTM training windows, estimate the class-1 residual mean and standard deviation, estimate the EWMA control limit, and calculate the reported class-1 false-positive rate. With w = 10, this yields 477 aligned class-1 predictions. The full ordered sequence is then processed for descriptive evaluation. Results are therefore described as calibration-inclusive, providing a controlled initial benchmark of false-positive behavior under the fitted configuration. The upstream caller-comparison labels serve as reference annotations based on the candidate-caller comparison performed during benchmark generation.

### 2.3 Phase I: sliding window construction and LSTM training

A sliding window converts the ordered genomic-window table into supervised samples. For sample t, the input contains the preceding w feature vectors and the target is F8 at sample t:


$$\mathrm{Input}:{\text{X}}_{\text{t}}=\left[{\text{x}}_{\text{t-w}},\ldots ,{\text{x}}_{t-1}\right]$$

$$\mathrm{Target}:{\text{y}}_{\text{t}}={\text{x}}_{t,8}$$

The input therefore contains only samples preceding the prediction target. The LSTM has L stacked layers with H hidden units per layer, followed by a fully connected layer mapping the final hidden state to one scalar. The mean squared error (MSE) over the class-1 training windows is minimized:


$${\hat{\text{y}}}_{\text{t}}={\text{f}}_{\text{LSTM}}\,\left({\text{X}}_{\text{t}};\theta \right)$$

$$\text{MSE}=\left(\frac{1}{{\text{N}}_{\text{IC}}-w}\right)\,\overset{{\text{N}}_{\text{IC}}}{\underset{\text{t}=\text{w}+1}{\sum }}{\left({\text{y}}_{\text{t}}-{\hat{\text{y}}}_{\text{t}}\right)}^{2}$$

The model was optimized with Adam (learning rate 0.001; batch size 32; maximum 200 epochs). A patience value of 15 was applied to training loss, providing a consistent stopping heuristic within the current benchmark. Seed 42 was set for NumPy and PyTorch. Deterministic GPU algorithms were not enforced; the seed supports repeatability, while deterministic settings can be enabled when bitwise-identical GPU execution is required.

### 2.4 Phase II: residual standardization

The fitted LSTM generates predictions for all aligned samples. The raw residual is the observed minus predicted scaled F8 value:


$${\text{e}}_{\text{t}}={\text{y}}_{\text{t}}-{\hat{\text{y}}}_{\text{t}}$$

Residuals are standardized using the sample mean and sample standard deviation estimated from the 477 aligned class-1 residuals:


$${\text{e}}_{\mathrm{std},t}=\frac{{\text{e}}_{\text{t}}-\mu_{e,\mathrm{IC}}}{\sigma_{e,\mathrm{IC}}}$$

$$\text{where}\,\mu_{e,\mathrm{IC}}=\left(\frac{1}{{\text{N}}_{\text{IC}}-w}\right)\,\overset{{\text{N}}_{\text{IC}}}{\underset{\text{t}=\text{w}+1}{\sum }}{\text{e}}_{\text{t}}$$

This operation centers and scales the class-1 residual sample by construction. The standardized residual is therefore treated as an empirical score, providing a basis for further characterization of its distributional and dependence properties using future validation data.

### 2.5 Phase III: EWMA control chart construction

For standardized residual e_*std*,t_, the EWMA statistic is computed recursively as:


$${\text{Z}}_{\text{t}}=\lambda \,{\text{⋅e}}_{\mathrm{std},t}+\left(1-\lambda \right)\,{\text{⋅Z}}_{t-1}$$

where λ∈ (0, 1) controls smoothing. The experiment code initializes Z_0_ = λe_*std*,0_. Smaller λ gives greater cumulative weight to earlier residuals; larger λ gives greater relative weight to the current residual. These weighting properties allow the realized detection delay to be evaluated empirically for each ordered sequence.

The bilateral limits are estimated empirically from the class-1 EWMA values. With sample standard deviation s(Z_I*C*_), half-width h = k × s(Z_I*C*_), and the default k = 3:


UCL=h,LCL=−h

$$\text{h}=\text{k⋅}\,s\,\left({\text{Z}}_{\text{IC}}\right),k=3$$

An alarm occurs when | Z_t_| > h. Because the limit is estimated from serially ordered, model-derived residuals and the same class-1 sequence used for fitting, the analysis reports benchmark-observed false-alarm behavior rather than assigning a nominal 0.27% probability or prespecified in-control average run length. Independent calibration can extend average-run-length evaluation.

### 2.6 Evaluation metrics

Reference class is converted to a binary target b_t_: class 1 is negative and classes 2–5 are positive. We report the following descriptive metrics on the aligned sequence:

False Positive Rate (FPR): the proportion of aligned class-1 samples signaled by the method.


$$\text{FPR}=\frac{\text{FP}}{\text{FP}+\mathrm{TN}}$$

False Negative Rate (FNR): the proportion of aligned class-2-to-5 samples not signaled by the method.


$$\text{FNR}=\frac{\text{FN}}{\text{FN}+\mathrm{TP}}$$

F1-score: the harmonic mean of precision and recall for the dispatch-positive class.


$$\mathrm{F1}=\frac{2\cdot \mathrm{Precision}\cdot \mathrm{Recall}}{\text{Precisio}\,n+\mathrm{Recall}}$$

Detection delay: the number of aligned samples between the first class-2-to-5 sample and the first alarm. A value of 0 denotes an alarm at that first sample.

Area under the receiver-operating-characteristic curve (AUC): discrimination across thresholds using | Z_t_| for EWMA methods and | e_*std*,t_| for the fixed-threshold LSTM comparator. Point estimates provide a baseline for future interval estimation.

## 3 Results

### 3.1 Benchmark and experimental setup

The benchmark comprises 1,510 ordered human-reference simulation windows, eight sequencing-derived features per window, and a reference optimal-caller code. Class 1 contains 487 samples; classes 2–5 contain 1,023 samples in total. The analysis begins from this derived feature table, isolating screening after upstream raw-read simulation, feature extraction, caller execution, and caller ranking generated the benchmark inputs. Results therefore characterize the screening stage conditional on the supplied table and labels.

The prespecified configuration used for the main comparison is shown in Table 1.

**TABLE 1:** Default hyperparameter configuration.

Hyperparameter	Value
Sliding window size (w)	10
LSTM hidden units	64
LSTM layers	2
Dropout rate	0.2
Batch size	32
Learning rate	0.001
Early stopping patience	15
EWMA smoothing parameter (λ)	0.05
Control limit multiplier (k)	3

Experiments were run with PyTorch 2 on a Windows workstation using seed 42. One LSTM run was evaluated for each listed setting. The ARIMA comparator used order (2, 1, 2), fitted on the class-1 F8 sequence and applied to the full sequence with the fitted parameters; the public script contains an expanding-refit procedure only as an exception fallback. The ARIMA results therefore provide a reproducible comparison for this prespecified implementation.

### 3.2 Baseline comparison

We compared the proposed screening statistic with two prespecified implementations:

(1) ARIMA–EWMA: An ARIMA (2,1,2) model fitted to the class-1 F8 sequence, followed by empirical residual standardization and EWMA monitoring.
(2) LSTM + fixed threshold: The same LSTM and standardized residuals without EWMA smoothing; an alarm occurs when the absolute standardized residual exceeds an empirical three-standard-deviation threshold. This comparison isolates the effect of smoothing within the evaluated implementation.

**FIGURE 2:**
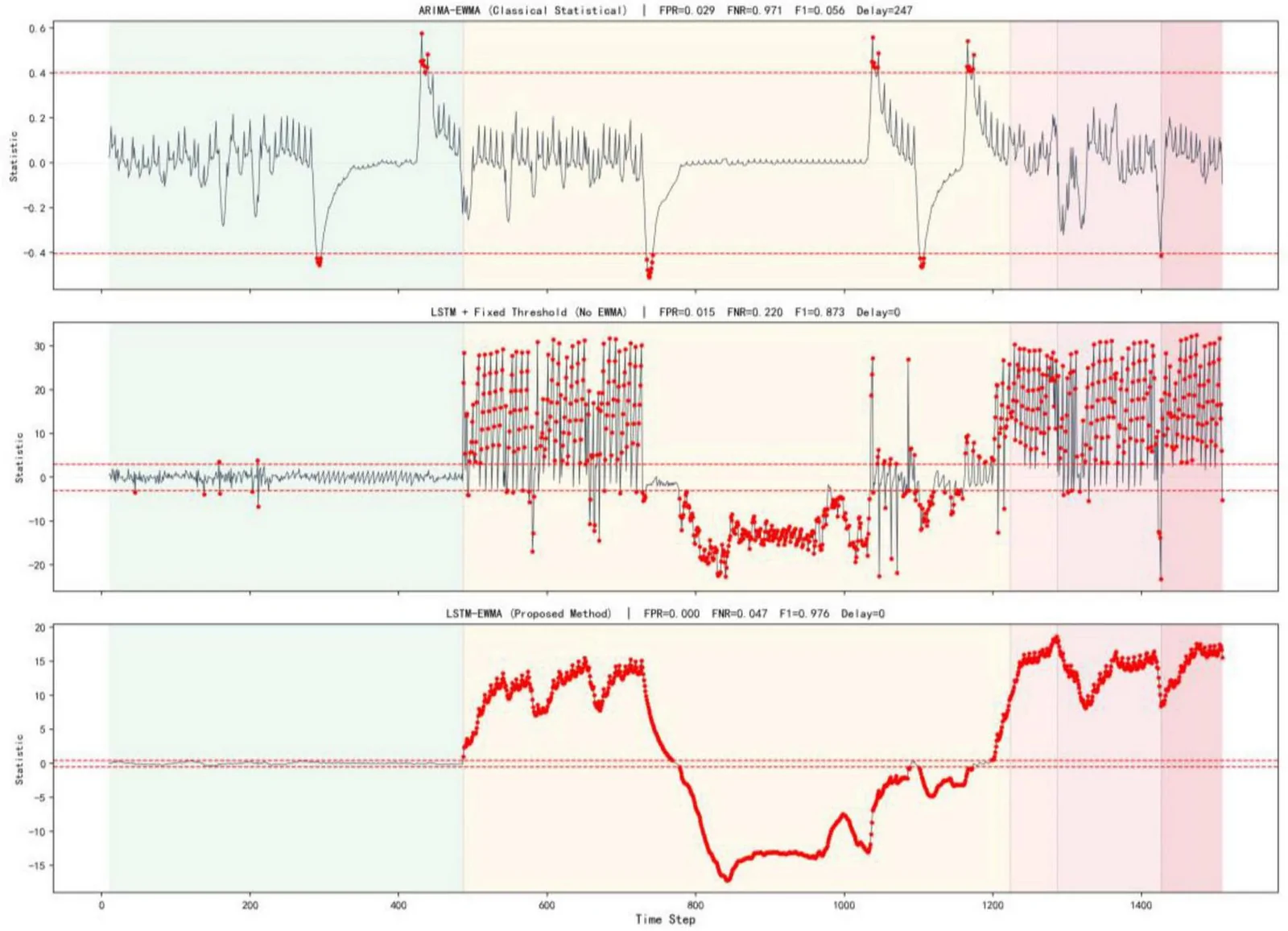
Descriptive control-chart comparison. **(Top)** ARIMA–EWMA. **(Middle)** LSTM + fixed threshold. **(Bottom)** LSTM–EWMA. Background colors identify the five reference caller classes; red markers denote alarms.

Figure 2 and Table 2 summarize one run on the fixed, calibration-inclusive benchmark. These values provide a descriptive within-benchmark comparison and a quantitative baseline for subsequent validation on independently generated sequencing data.

**TABLE 2:** Descriptive detection metrics for the fixed benchmark.

Method	FPR	FNR	F1-score	Detection delay
ARIMA–EWMA	0.0294	0.9707	0.0562	247
LSTM + fixed threshold	0.0147	0.2199	0.8731	0
LSTM–EWMA (Proposed)	0.0000	0.0469	0.9760	0

LSTM–EWMA produced no observed alarms among the 477 aligned class-1 points (FPR = 0.0000). This calibration-inclusive observation reflects that those points informed scaling, model fitting, residual standardization, and the control limit. It characterizes false-alarm behavior under the fitted configuration and provides a benchmark for independent evaluation. The corresponding observed FPR values were 0.0294 for ARIMA–EWMA and 0.0147 for LSTM + fixed threshold.

For class-2-to-5 samples, observed FNR was 0.0469 for LSTM–EWMA, 0.2199 for LSTM + fixed threshold, and 0.9707 for the prespecified ARIMA–EWMA implementation. Within this sequence, the prespecified ARIMA configuration provides a defined comparator for LSTM-based residual modeling; other orders and fitting strategies remain directions for extension.

Observed F1-score was 0.9760 for LSTM–EWMA, 0.8731 for LSTM + fixed threshold, and 0.0562 for ARIMA–EWMA. These point estimates provide a descriptive baseline for future repeated simulations, interval estimation, and statistical comparison.

The first class-2-to-5 sample was signaled without an aligned-sample delay by both LSTM implementations. The ARIMA–EWMA implementation first signaled 247 samples later. This observed delay provides a sequence-level reference for future evaluation across additional data-generating conditions.

### 3.3 Sensitivity analysis

One-factor sensitivity analyses varied λ or w while retaining the remaining implementation choices. Each setting was evaluated once with seed 42; the tables report observed values for this controlled run and provide a basis for repeated-seed analysis.

#### 3.3.1 EWMA smoothing sensitivity

**FIGURE 3:**
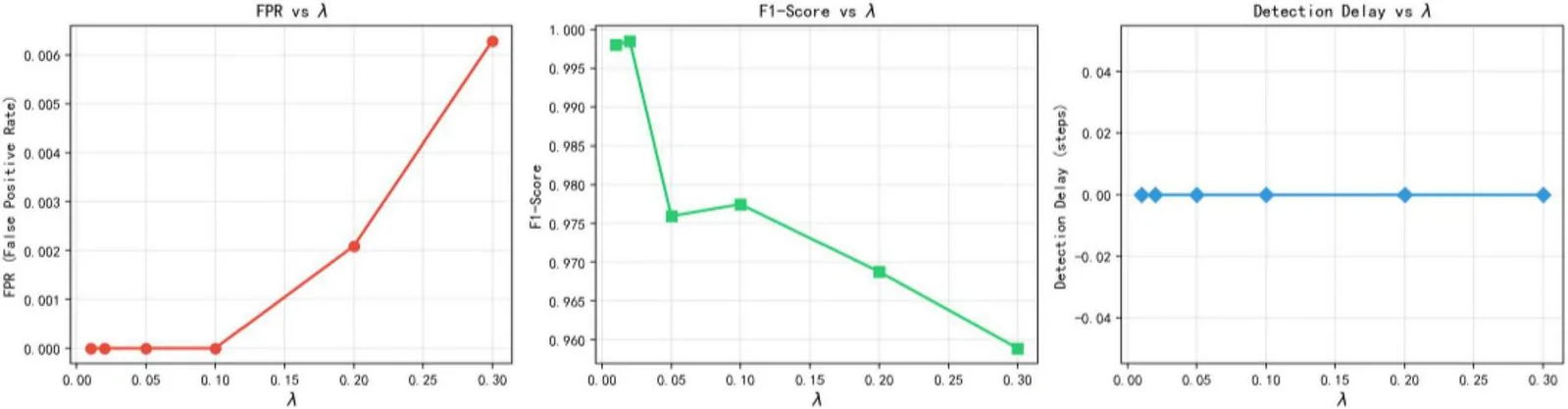
One-run sensitivity analysis for λ. **(Left)** False Positive Rate (FPR). **(Center)** F1-score. **(Right)** Detection delay.

Figure 3 and Table 3 reports results for λ∈ {0.01, 0.02, 0.05, 0.10, 0.20, 0.30} using the same fitted default-window LSTM residual sequence.

**TABLE 3:** One-run sensitivity analysis for the Exponentially Weighted Moving Average (EWMA) smoothing parameter λ.

λ	FPR	FNR	F1-score	Detection delay
0.01	0.0000	0.0039	0.9980	0
0.02	0.0000	0.0029	0.9985	0
0.05	0.0000	0.0469	0.9760	0
0.10	0.0000	0.0440	0.9775	0
0.20	0.0021	0.0596	0.9688	0
0.30	0.0063	0.0762	0.9589	0

For λ from 0.01 to 0.10, observed FPR was 0 and F1-score ranged from 0.9760 to 0.9985. The lowest observed FNR (0.0029) and highest observed F1-score (0.9985) occurred at λ = 0.02, not at the default λ = 0.05. At λ = 0.20 and 0.30, observed FPR and FNR increased. These six points identify the best observed setting and a starting range for broader parameter evaluation.

All evaluated λ values had observed delay 0 on this sequence; sensitivity therefore distinguishes them through FPR, FNR, and F1-score. λ = 0.05 remains the prespecified default, while future independent calibration can refine context-specific selection.

#### 3.3.2 Sliding-window sensitivity

**FIGURE 4:**
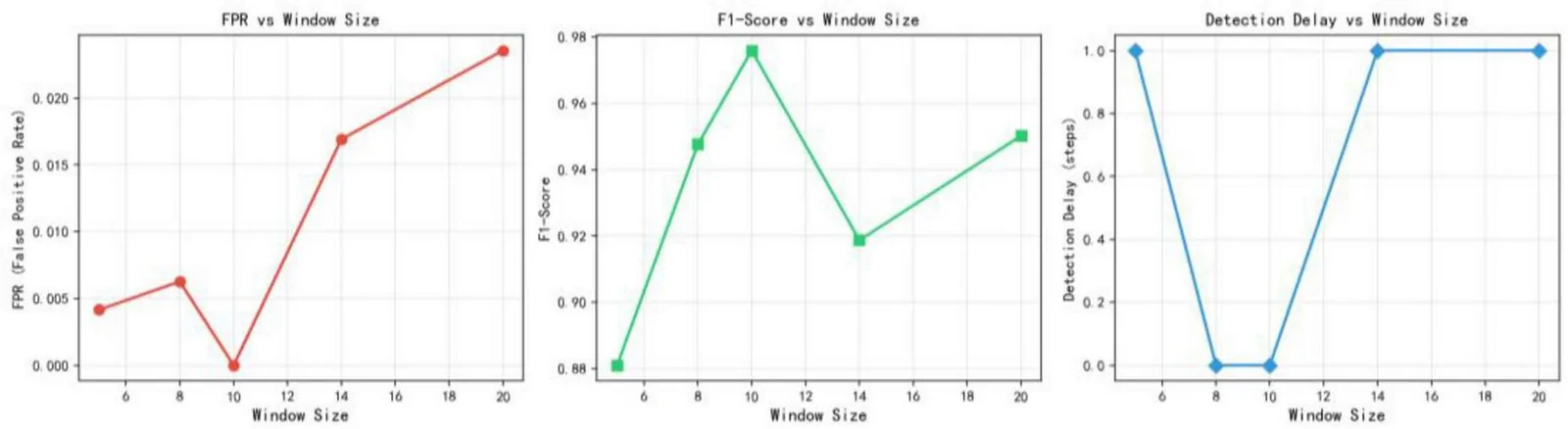
One-run sensitivity analysis for window size. **(Left)** False Positive Rate (FPR). **(Center)** F1-score. **(Right)** Detection delay.

Figure 4 and Table 4 report results for window sizes ∈ {5, 8, 10, 14, 20}; the LSTM was retrained once for each setting.

**TABLE 4:** One-run sensitivity analysis for sliding-window size.

Window size	FPR	FNR	F1-score	Detection delay
5	0.0041	0.2111	0.8810	1
8	0.0063	0.0968	0.9477	0
10	0.0000	0.0469	0.9760	0
14	0.0169	0.1437	0.9187	1
20	0.0236	0.0850	0.9503	1

Among the five evaluated settings, w = 10 had the highest observed F1-score (0.9760) and the only observed FPR of 0.0000. The remaining F1-scores ranged from 0.8810 to 0.9503. These results identify the best observed setting in this benchmark run and provide a starting point for broader evaluation of window-size effects across independent sequences and data-generating conditions.

#### 3.3.3 ROC analysis

**FIGURE 5:**
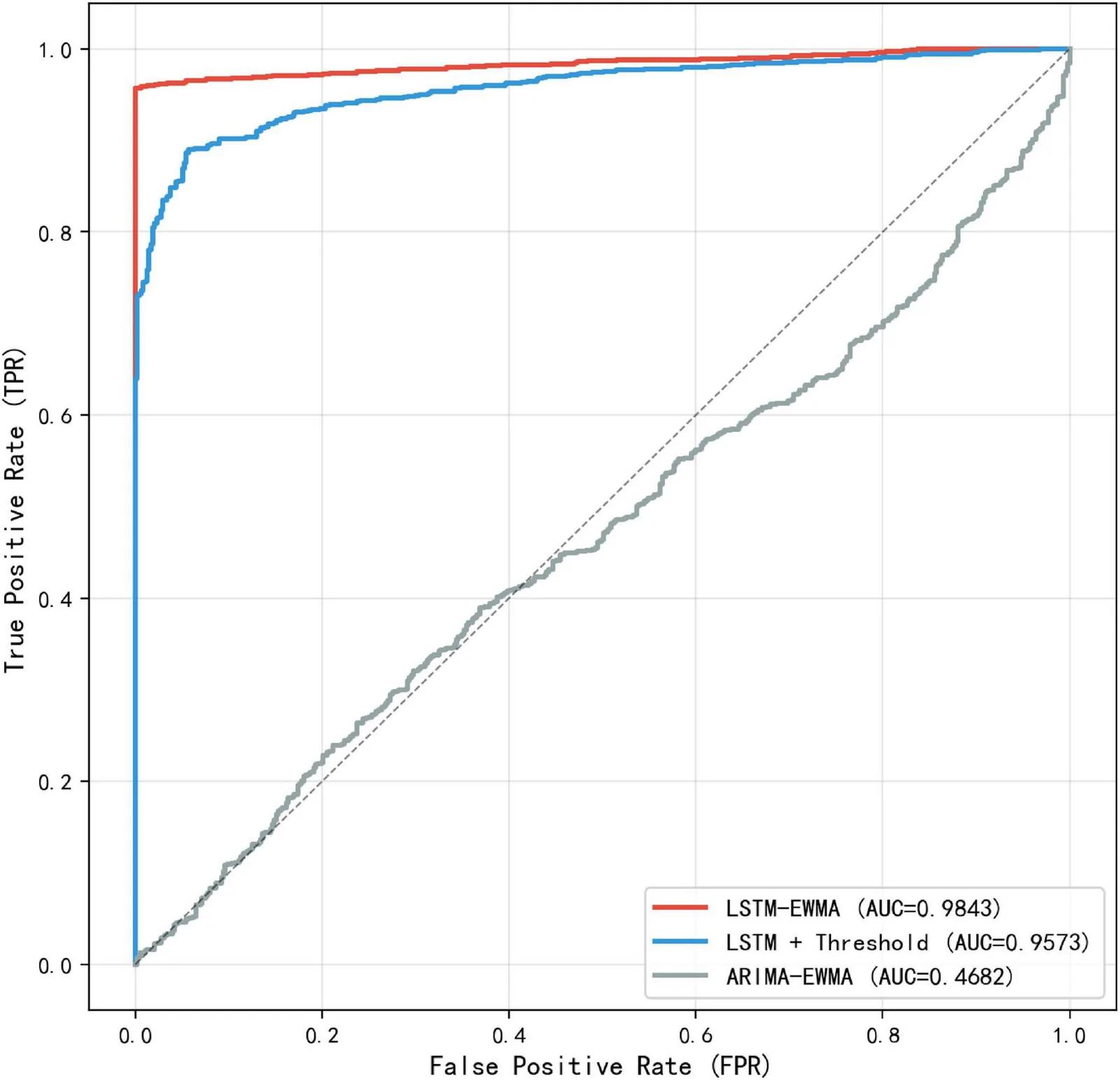
Calibration-inclusive ROC curves for the three evaluated implementations.

Figure 5 and Table 5 show ROC curves and AUC values calculated on the same aligned benchmark sequence used for model calibration.

**TABLE 5:** Calibration-inclusive area under the receiver-operating-characteristic curve (AUC) values for the fixed benchmark.

Method	AUC
LSTM–EWMA (proposed)	0.9843
ARIMA–EWMA	0.4682
LSTM + fixed threshold	0.9573

Observed AUC was 0.9843 for LSTM–EWMA, 0.9573 for LSTM + fixed threshold, and 0.4682 for ARIMA–EWMA. These calibration-inclusive values describe rank separation on the fixed sequence and provide a quantitative reference for future independent generalization assessment. The ARIMA value corresponds to the prespecified ARIMA (2,1,2) implementation.

### 3.4 Component comparison

**FIGURE 6:**
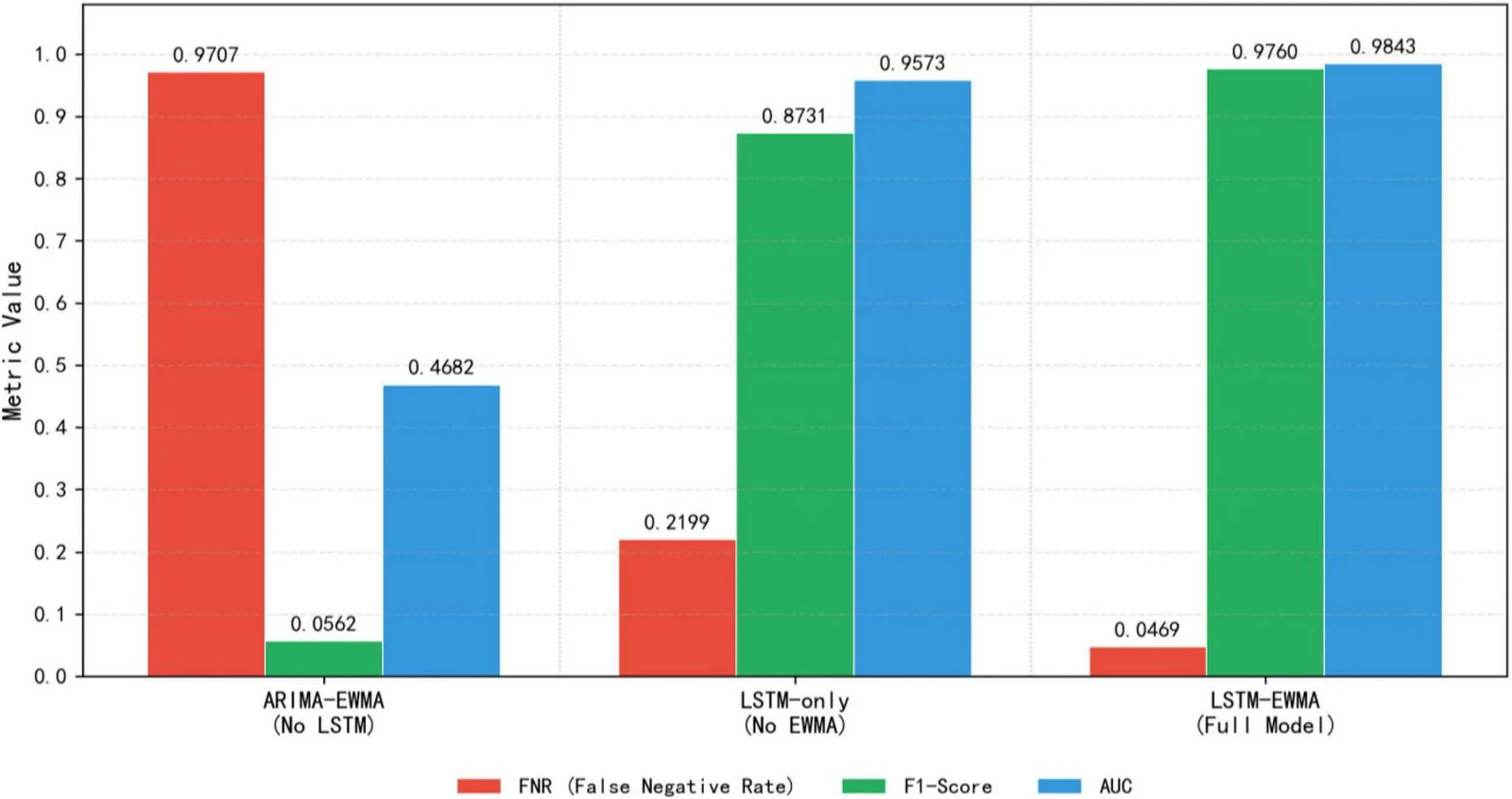
Component comparison. Bars report False Negative Rate (FNR), F1-score, and area under the receiver-operating-characteristic curve (AUC) for the three configurations. False Positive Rate (FPR) is reported in Table 6 but is not plotted.

The three configurations in Figure 6 and Table 6 compare the two components within the same benchmark and seed:

(1) ARIMA–EWMA: Replaces the LSTM predictor with the prespecified ARIMA (2,1,2) implementation while retaining EWMA.
(2) LSTM + fixed threshold: Retains LSTM residuals and removes EWMA smoothing.
(3) LSTM–EWMA: Retains both the LSTM predictor and EWMA smoothing.

**TABLE 6:** Descriptive component comparison on the fixed benchmark.

Method	LSTM	EWMA	FPR	FNR	F1-score	Delay
ARIMA–EWMA	N	Y	0.0294	0.9707	0.0562	247
LSTM + fixed threshold	Y	N	0.0147	0.2199	0.8731	0
LSTM–EWMA	Y	Y	0.0000	0.0469	0.9760	0

Replacing the LSTM with the prespecified ARIMA implementation increased observed FNR from 0.0469 to 0.9707, reduced F1-score from 0.9760 to 0.0562, and increased delay from 0 to 247. Within this benchmark, the LSTM residual score separated the supplied binary caller labels more effectively than the selected ARIMA residual score. This contrast provides a basis for testing the LSTM contribution across other ARIMA orders, fitting strategies, datasets, and caller sets.

Removing EWMA smoothing increased observed FPR from 0.0000 to 0.0147 and FNR from 0.0469 to 0.2199, while AUC changed from 0.9843 to 0.9573. The result is consistent with a benefit from smoothing on this residual sequence and provides an initial component-level indication for subsequent repeated ablations and external-data evaluation.

## 4 Discussion

Efficient triage of genomic regions is important for large-scale sequencing studies because applying a region-specific variant-caller recommendation procedure exhaustively across all genomic windows can impose substantial computational demands. This study addresses this challenge by representing ordered genomic regions as a computational sequence and integrating an LSTM predictive model with EWMA control charts. Within the resulting framework, prediction residuals are monitored to identify windows that may benefit from specialized downstream evaluation. Unflagged windows remain on the prespecified default processing path, whereas alarmed windows are dispatched to the more resource-intensive recommendation procedure. This design provides a practical way to concentrate computational resources on regions whose feature patterns depart from the learned baseline.

The same principle may be relevant to microbial genomes, host-associated microbiomes, and metagenomic assemblies, in which variation in sequence composition, repetitive content, local coverage, taxonomic diversity, and organismal abundance can affect the suitability of a uniform variant-calling workflow. The present experiments use simulated sequencing data derived from the human reference genome, providing a controlled initial benchmark with consistently defined region-level reference labels. This setting enables the dispatch mechanism to be evaluated independently and establishes a methodological foundation for subsequent studies involving microbial and metagenomic data. Building on this foundation, future validation across diverse microbial taxa, community compositions, abundance profiles, sequencing platforms, and biologically characterized host-associated systems can further assess the transferability and practical value of the framework.

## 5 Conclusion

In this paper, we introduced a dynamic dispatch framework that integrates Long Short-Term Memory (LSTM) networks with Exponentially Weighted Moving Average (EWMA) control charts to reduce the computational bottleneck of region-specific variant calling. By constructing the LSTM hypothesis exclusively on in-control (IC) data–representing simple genomic regions–and applying EWMA smoothing to standardized prediction residuals, the method detects complex regions that may require specialized processing while maintaining low false alarm rates.

Experiments on simulated sequencing data derived from the human reference genome support the feasibility of the proposed approach. The LSTM-EWMA framework achieves a false positive rate of 0.0000, a false negative rate of 0.0469, an F1-score of 0.9760, and an AUC of 0.9843, outperforming ARIMA-EWMA and LSTM-only baselines on this benchmark. Sensitivity analysis confirms the method’s robustness across a range of hyperparameter values, with consistent near-optimal performance for lambda in [0.01, 0.10] and w in Li and Durbin (2009), Treangen and Salzberg (2011). The ablation study demonstrates the contribution of both the LSTM hypothesis and the EWMA smoothing component, validating the advantage of combining deep learning with statistical process control for a computational dispatch workflow.

Several directions for future work remain. First, future research will extend the framework to multivariate EWMA charts to monitor multiple genomic characteristics, such as local GC content, repetitive elements, coverage depth, and candidate horizontal gene transfer signatures. Second, adaptive lambda selection based on the evolving residual distribution could dynamically optimize the detection-delay trade-off across different sequencing contexts. Third, incorporating attention mechanisms or transformer architectures into the predictive hypothesis could improve long-range dependency modeling for extended structural variants. Finally, validation on real microbial, environmental, host-associated metagenomic, and multi-species benchmark datasets across different sequencing platforms will be necessary to establish the generalizability of the proposed methodology for microbiology and microbial symbiosis research.

## Data Availability

The source code supporting this study is publicly available at https://github.com/Icarus200110/Lstm-EWMA. The benchmark data are available from the corresponding author upon reasonable request.
